# FOXA1 in Ovarian Cancer: A Potential Therapeutic Target to Enhance Immunotherapy Efficacy

**DOI:** 10.3390/ijms27031194

**Published:** 2026-01-24

**Authors:** Taewan Kim, Jaesung Ryu, Hyejeong Kong, Beamjun Park, Kwangseock Kim, Eunjung Yang, Taesung Ahn, Seob Jeon

**Affiliations:** 1 Future Innovation Medical Research Center, Soonchunhyang University Cheonan Hospital, Cheonan 31151, Republic of Korea; ktwdreem@naver.com (T.K.); kimks5005gt@gmail.com (K.K.); 2 Department of Medical Life Science, Soonchunhyang University, Asan 31538, Republic of Korea; rjs950826@gmail.com (J.R.); angelkonghj@gmail.com (H.K.); cool373867@gmail.com (B.P.); 3 Department of Gynecology, College of Medicine, Soonchunhyang University Cheonan Hospital, Cheonan 31151, Republic of Korea; 117652@schmc.ac.kr; 4 Department of Colorectal Surgery, College of Medicine, Soonchunhyang University Cheonan Hospital, Cheonan 31151, Republic of Korea; eyetoeye@schmc.ac.kr

**Keywords:** ovarian cancer, FOXA1, forkhead box A1, oncogene, chemoresistance, immune checkpoint inhibitor, biomarker

## Abstract

This study aimed to elucidate the oncogenic role of FOXA1(forkhead box A1) in ovarian cancer and to evaluate its potential as both a therapeutic target and a diagnostic biomarker. We further investigated whether FOXA1 inhibition could enhance responsiveness to immune checkpoint blockade and overcome chemoresistance. A total of seventy-six ovarian tissue samples were analyzed, including nine normal, thirty-four benign, and thirty-three malignant specimens. IHC (immunohistochemistry) staining was performed to assess FOXA1 expression and its correlation with tumor stage. Functional studies were conducted using FOXA1 siRNA in SK-OV3 and HEYA8 cell lines. Changes in cell proliferation, migration, invasion, and wound-healing ability were evaluated following FOXA1 silencing. Quantitative RT-PCR was used to measure the expression of FOXA1 and EMT (epithelial–mesenchymal transition)-related genes. The effects of FOXA1 inhibition on sensitivity to carboplatin and the immune checkpoint inhibitor atezolizumab were also examined. IHC analysis revealed significant differences in FOXA1 expression among normal, benign, and malignant tissues, with levels correlating with tumor stage. FOXA1 silencing significantly reduced proliferation and decreased migration and invasion by 60–80%, accompanied by marked downregulation of EMT-related genes. Moreover, FOXA1 inhibition enhanced atezolizumab responsiveness and reduced carboplatin resistance in ovarian cancer cells. In summary, FOXA1 acts as an oncogenic driver in ovarian cancer, promoting proliferation, invasion, and EMT activation. Its overexpression correlates with disease progression, supporting its potential as a biomarker and therapeutic target. Targeting FOXA1 could enhance immunotherapy efficacy and help overcome chemoresistance in ovarian cancer.

## 1. Introduction

Ovarian cancer is one of the cancer types with a high recurrence rate, with approximately 10% recurrence for stage I ovarian cancer, while the recurrence rates for stages III and IV rise to approximately 70–80% [1]. This is primarily due to the acquisition of resistance to platinum-based chemotherapy drugs, which are commonly used in ovarian cancer. For this reason, the 5-year survival rates for stage III and IV ovarian cancer are only 26% and 14%, respectively [2]. Moreover, while numerous oncogenes have been identified in other cancer types, leading to many therapeutic targets, the number of such targets for ovarian cancer is limited, and research on the functional roles of oncogenes in this context is relatively sparse [3,4]. In our previous study, NGS analysis of ovarian cancer patient tissues and blood samples revealed overexpression of miR-1290 in these patients [5]. To elucidate the role of FOXA1, one of the target genes of miR-1290, in ovarian cancer, this study was conducted. FOXA1 and FOXA2 belong to the forkhead box A family of proteins and are primarily known for their roles in early embryonic development, organ development, metabolic regulation, and cancer. FOXA1 and FOXA2 regulate glucose and lipid metabolism and play important roles in a variety of metabolic diseases and cancer [6]. In cancer, they play a role in tumor development, growth, metastasis, and drug resistance. The two transcription factors act complementarily and sometimes competitively with each other. For example, FOXA1 affects insulin secretion, while FOXA2 is involved in glucagon secretion. They play opposing roles in metabolic regulation [7]. In prostate cancer, FOXA1 and FOXA2 interact with the androgen receptor (AR) and are associated with prostate cancer development and drug resistance [8]. Increased expression of FOXA1 contributes to the growth and metastasis of cancer cells. In breast cancer, FOXA1 interacts with the estrogen receptor (ER) to influence the growth and drug resistance of breast cancer cells [9]. FOXA2 intervenes in epithelial–mesenchymal transition (EMT) of breast cancer and inhibits metastasis [10]. In liver cancer, FOXA1 and FOXA2 regulate sex differences in liver cancer and play a role in the development and progression of hepatocellular carcinoma (HCC). FOXA2 contributes to metabolic regulation in the liver, and its lower expression is associated with increased cancer aggressiveness. [11] Taken together, these findings indicate that FOXA1 plays diverse roles across different types of cancer. In our previous study, we observed that FOXA1 was overexpressed in ovarian cancer tissues, which led us to investigate its specific role in ovarian cancer progression. [5]

In this study, functional assays—including proliferation, migration, and invasion tests—demonstrated that FOXA1 promotes the progression of ovarian cancer. Elevated expression of the FOXA1 gene in ovarian cancer cells was correlated with more aggressive cellular behavior, particularly through the upregulation of epithelial–mesenchymal transition (EMT)-related genes. Immunohistochemical (IHC) analysis revealed statistically significant differences in FOXA1 expression among normal, benign, and malignant ovarian tissues, and further confirmed stage-specific variations within the tumor group. Furthermore, chemoresistance assays using carboplatin showed that higher FOXA1 expression was associated with increased drug resistance, whereas FOXA1 suppression reduced resistance. Notably, inhibition of FOXA1 enhanced responsiveness to the immune checkpoint inhibitor atezolizumab, suggesting that FOXA1 plays a crucial role in modulating immunotherapeutic efficacy in ovarian cancer.

## 2. Results

### 2.1. Confirmed the Inhibition Efficiency of FOXA1 by siRNA in Ovarian Cancer Cells

**Figure 1:**
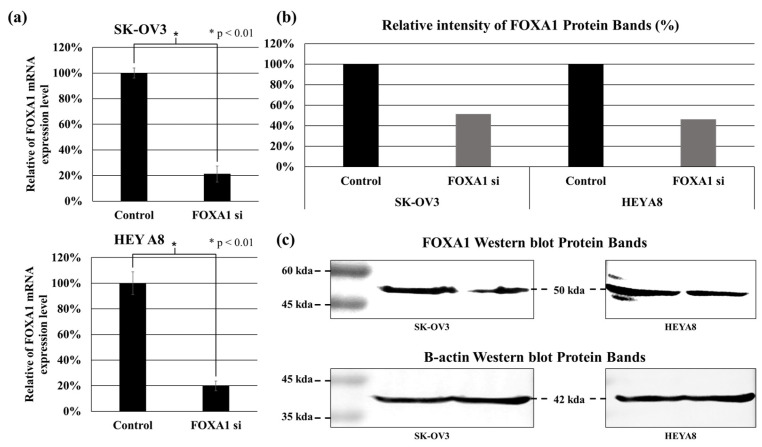
Verification of mRNA and protein level expression in FOXA1 siRNA-treated ovarian cancer cells. (**a**) FOXA1 mRNA expression after FOXA1 siRNA transfection. (**b**) Quantification of FOXA1 protein expression. (**c**) Representative Western blot images of FOXA1 and β-actin after FOXA1 siRNA transfection.

We confirmed the knockdown of FOXA1 by siRNA transfection in two types of ovarian cancer cell lines (SK-OV3, HEY A8). Figure 1 shows a graph comparing RNA and protein expression in FOXA1 knockdown cells, and all cell lines showed a 20% or less suppression of expression in FOXA1 knockdown cells compared to control (*p* < 0.01). Based on these results, we conducted cell proliferation analysis, Transwell migration assay, Transwell invasion assay, and wound healing assay experiments. Real-time polymerase chain reaction was used to analyze FOXA1 mRNA expression in the ovarian cancer cell lines SK-OV3 and HEYA8 treated with FOXA1 siRNA. FOXA1 mRNA levels were reduced by approximately 80% in FOXA1 siRNA-treated cells compared with control cells in both SK-OV3 and HEYA8 cell lines (Figure 1a). Western blot analysis was used to assess FOXA1 protein expression in the ovarian cancer cell lines SK-OV3 and HEYA8 following FOXA1 siRNA treatment. Protein loading and normalization were controlled by using β-actin as an internal loading control. FOXA1 protein expression levels were normalized to the corresponding β-actin signals to ensure equal protein loading across samples. FOXA1 protein levels were reduced by approximately 50% in FOXA1 siRNA-treated cells compared with control cells in both SK-OV3 and HEYA8 cell lines (Figure 1b,c).

### 2.2. Effect of FOXA1 Silencing on Cell Proliferation in Ovarian Cancer Cells

**Figure 2:**
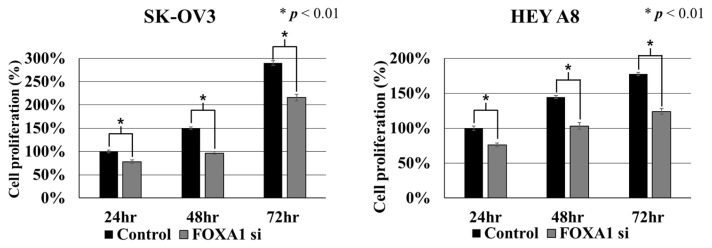
Cell proliferation analysis to silence of FOXA1 in ovarian cancer cells. FOXA1 silencing in SK-OV3 and HEYA8 ovarian cancer cell lines reduces cell proliferation compared to control. (* *p* < 0.01).

To evaluate the effect of FOXA1 suppression on cell growth, a cell proliferation assay was performed in SK-OV3 and HEYA8 cells at 24, 48, and 72 h after siRNA transfection (Figure 2). EZ-Cytox absorbance measurements showed that FOXA1 knockdown significantly reduced the proliferation rate compared with the control in both cell lines at all time points (*p* < 0.01). These results indicate that FOXA1 contributes to the proliferative capacity of ovarian cancer cells. All experiments were performed in triplicate.

### 2.3. Silence of FOXA1 Can Regulate Metastasis-Related Functions of Cancer Cells

**Figure 3:**
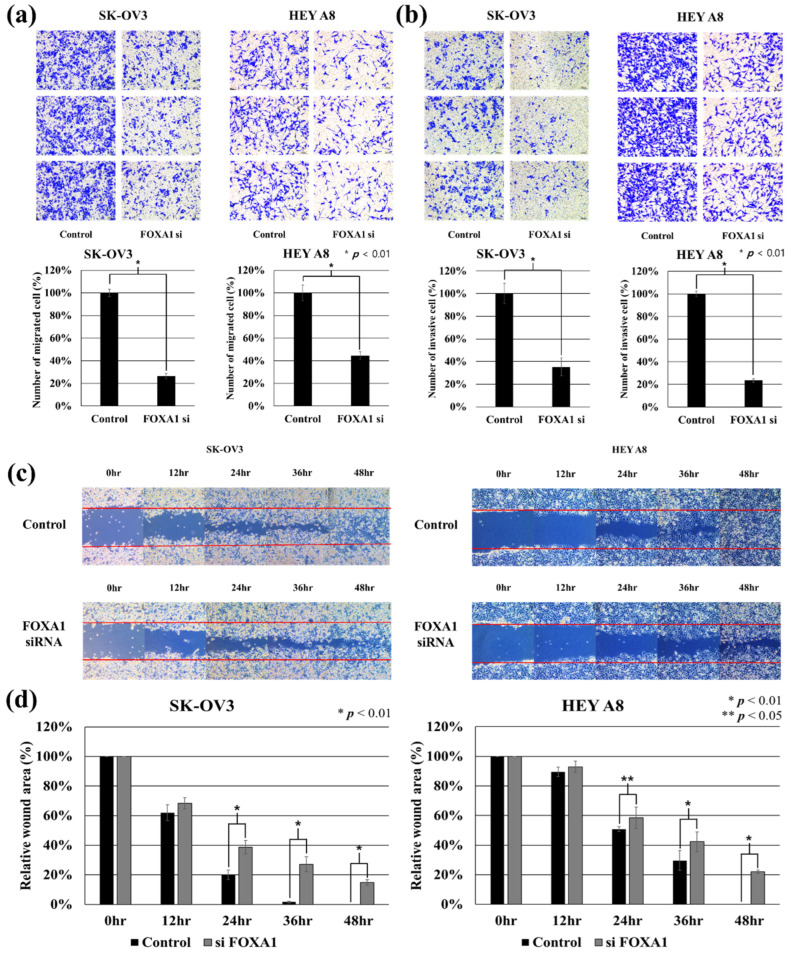
Cell migration, invasion, and wound healing assay to silence of FOXA1 in ovarian cancer cells. (**a**) FOXA1 si decreased the migration ability of ovarian cancer cells (Left: SK-OV3, Right: HEYA8). (**b**) FOXA1 si decreased the invasion ability of ovarian cancer cells (Left: SK-OV3, Right: HEYA8). (**c**) Representative images of the wound healing assay at 0, 12, 24, 36, and 48 h time points (Left: SK-OV3, Right: HEYA8). (**d**) The quantitative evaluation and statistical analysis of wound area percentage in wound healing assay measured by Image J software. Results are expressed as mean ± SD of five experiments (Left: SK-OV3, Right: HEYA8).

We performed migration, invasion, and wound healing assays to evaluate the association between FOXA1 inhibition and metastatic function in ovarian cancer cells (Figure 3). The silence of FOXA1 reduced the migration cells of SK-OV3 (about 73%) and HEYA8 (about 55%) by more than half compared to control (* *p* < 0.01). Furthermore, the silence of FOXA1 reduced the invasion cells of SK-OV3 (about 65%) and HEYA8 (about 77%) by more than half compared to control (* *p* < 0.01). The silence of FOXA1 significantly reduced the wound healing function of SK-OV3 and HEYA8. The wound closure area of FOXA1 siRNA-transfected SK-OV3 and HEYA8 showed a significant difference compared to control at 24 h, 36 h, and 48 h after wound scratch (SK-OV3 (24, 36, 48 h: * *p* < 0.01)), (HEYA8 (24 h: ** *p* < 0.05, 36, 48 h: * *p* < 0.01)). These wound healing assay results support the previously performed migration and invasion assay results. These results imply that elevated FOXA1 expression might be linked to increased metastatic activity in ovarian cancer cells.

### 2.4. Association of FOXA1 Expression with the Expression of EMT Genes

To determine if FOXA1 expression in ovarian cancer cell lines makes them more susceptible to EMT, we treated the ovarian cancer cell lines with siRNA and then used real-time PCR to determine if there was a change in the expression of EMT genes.

**Figure 4:**
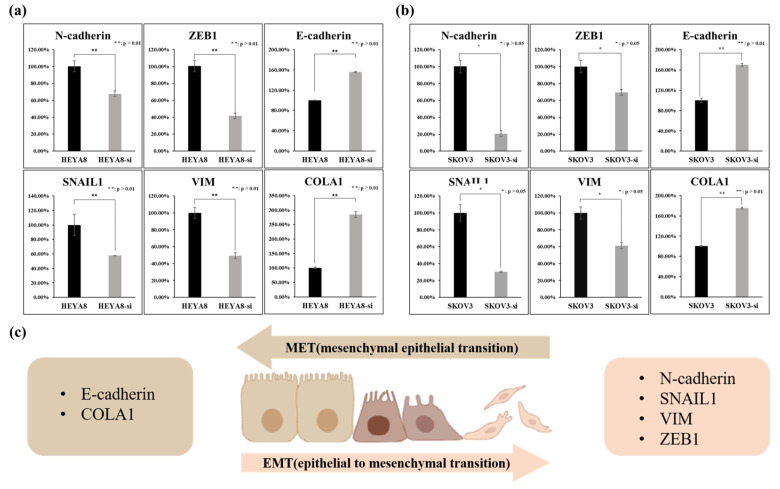
Comparison of EMT gene expression after treatment with FOXA1 siRNA. (**a**) Changes in EMT gene expression in HEYA8. (**b**) Changes in EMT gene expression in SK-OV3. (**c**) Schematic representation of EMT and MET processes and their key molecular markers.

FOXA1 expression in ovarian cancer shows an association with EMT-related patterns, and FOXA1 knockdown alters EMT and MET marker expression in HEYA8 and SK-OV3 cells. Expression levels of EMT markers (N-cadherin, VIM, SNAIL1, ZEB1), the MET marker COLA1, and E-cadherin were assessed by PCR in HEYA8 ovarian cancer cells after treatment with FOXA1 siRNA. Knockdown of FOXA1 resulted in significant downregulation of key EMT markers: N-cadherin expression decreased to 68%, VIM to 49%, SNAIL1 to 58%, and ZEB1 to 42% of control levels. Conversely, the MET marker COLA1 and E-cadherin were upregulated, with their expression increasing to 284% (COLA1) and 155% (E-cadherin) (Figure 4a). Expression levels of EMT markers (N-cadherin, SNAIL1, ZEB1, VIM) were analyzed by PCR in SKOV3 ovarian cancer cells following FOXA1 siRNA treatment. FOXA1 knockdown resulted in significant downregulation of these EMT markers: N-cadherin expression decreased to 21%, SNAIL1 to 30%, VIM to 61%, and ZEB1 to 60% of control levels. Conversely, the MET marker COLA1 and E-cadherin were upregulated, with their expression increasing to 175% (COLA1) and 170% (E-cadherin, Figure 4b). These findings are consistent with the changes observed in EMT-related gene (N-cadherin, SNAIL1, VIM, ZEB1, E-cadherin, COLA1) expression following FOXA1 suppression in SKOV3 and HEYA8 cells, further supporting the role of FOXA1 in promoting epithelial–mesenchymal transition (EMT) in ovarian cancer cells (Figure 4c).

### 2.5. Effect of FOXA1 Silencing on Chemoresistance to Carboplatin and Atezolizumab in SK-OV3 and HEYA8 Ovarian Cancer Cells

**Figure 5:**
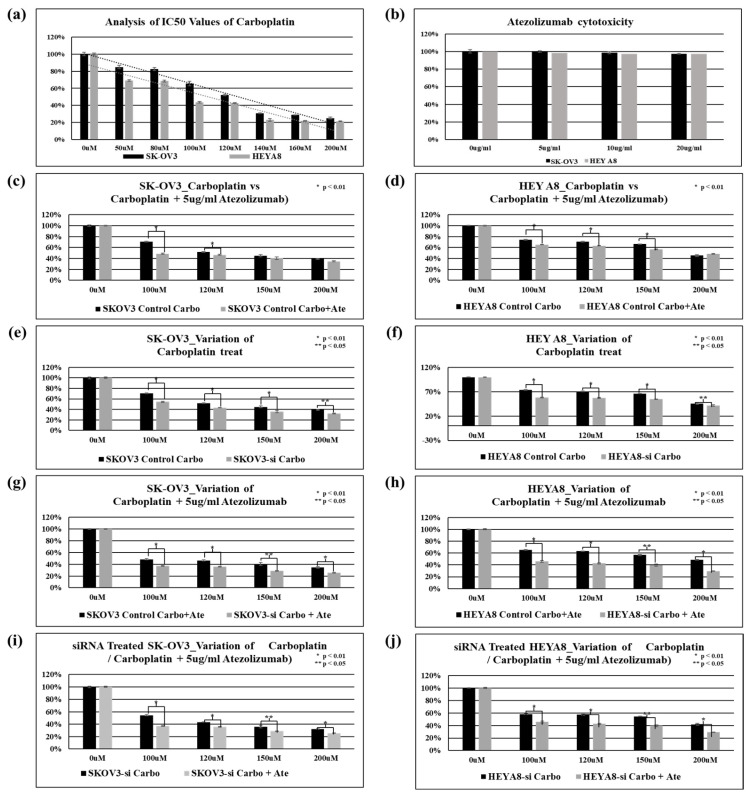
Reduction in chemoresistance following FOXA1 Gene Knockdown. (**a**) Determining IC50 values using carboplatin. (**b**) Atezolizumab cytotoxicity test in SK-OV3 and HEY A8 cells. (**c**,**d**) Cell viability of SK-OV3 and HEYA8 ovarian cancer cells following carboplatin treatment with or without atezolizumab. (**e**,**f**) Cell viability of SK-OV3 and HEYA8 ovarian cancer cells following carboplatin treatment with or without FOXA1 knockdown. (**g**,**h**) Cell viability of SK-OV3 and HEYA8 ovarian cancer cells following combined treatment with carboplatin and atezolizumab, with or without FOXA1 knockdown. (**i**,**j**) Comparison of cell viability in FOXA1-silenced SK-OV3 and HEYA8 cells treated with carboplatin alone or in combination with atezolizumab.

Cell viability and IC50 values following carboplatin treatment were assessed in SK-OV3 and HEYA8 ovarian cancer cell lines. The IC50 value of carboplatin was 125.48 μM in SK-OV3 cells and 102.67 μM in HEYA8 cells (Figure 5a). Treatment with the immune checkpoint inhibitor atezolizumab alone did not result in a significant reduction in cell viability in either cell line, indicating limited cytotoxic effects when used as monotherapy (Figure 5b). Cell viability following treatment with carboplatin alone or in combination with atezolizumab was evaluated in SK-OV3 and HEYA8 cells. Compared with control cells, carboplatin monotherapy or combined treatment with atezolizumab resulted in a significant reduction in cell viability at specific concentration ranges (SK-OV3: 100 and 120 μM; HEYA8: 100, 120, and 150 μM) (Figure 5c,d). Cell viability following carboplatin treatment was then evaluated in SK-OV3 and HEYA8 cells with or without FOXA1 knockdown. FOXA1 silencing significantly reduced cell viability compared with control cells, indicating decreased chemoresistance after carboplatin treatment (Figure 5e,f). In addition, cell viability following combined treatment with carboplatin and atezolizumab was compared in SK-OV3 and HEYA8 cells with or without FOXA1 knockdown. FOXA1 silencing significantly reduced cell viability compared with control cells, indicating decreased chemoresistance following combination treatment (Figure 5g,h). Furthermore, in FOXA1-knockdown SK-OV3 and HEYA8 cells, cell viability was compared between carboplatin monotherapy and combined carboplatin plus atezolizumab treatment. The combination treatment significantly reduced cell viability compared with carboplatin alone, further demonstrating reduced chemoresistance (Figure 5i,j).

### 2.6. Clinical and Pathological Characteristics of Patients

To investigate whether the expression levels of FOXA1 in ovarian cancer patients were associated with clinical information (age, tumor classification, survival, BRCA mutation, platinum), comparison was made using immunohistochemical staining (Table 1).

**Figure 6:**
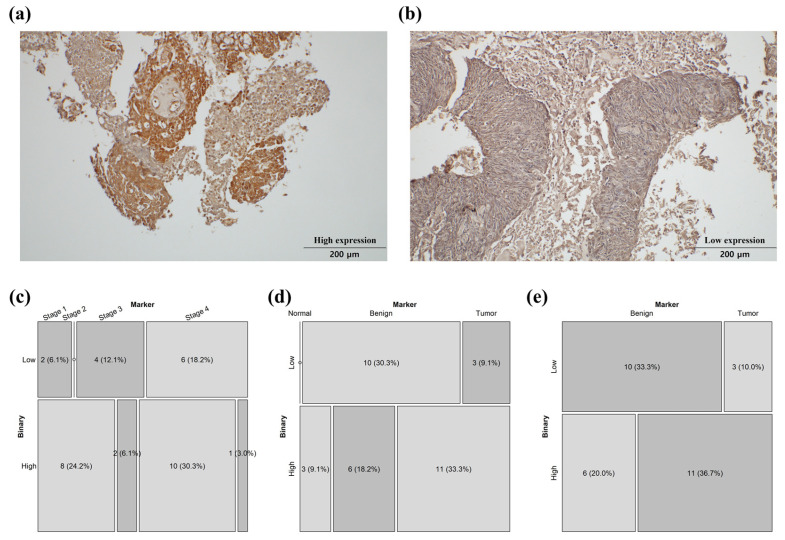
Immunohistochemical analysis of FOXA1 expression in ovarian cancer patient tissue samples. (**a**) High expression of FOXA1. (**b**) Low expression of FOXA1. (**c**) Malignant tumor patients stratified by stage (I–IV) (*p* < 0.05). (**d**) Patients under fifty years old stratified into normal, benign, and tumor groups (*p* < 0.05). (**e**) Patients under fifty years old stratified by benign vs. tumor only (*p* < 0.05).

The immunohistochemical (IHC) staining results for FOXA1 were evaluated by a pathologist using the most current interpretation criteria (High/Low; Figure 6a,b) [12]. To account for potential differences in ovarian function between pre- and post-menopausal women, the samples were divided into two cohorts: patients under fifty years of age and all patients combined [13,14]. Statistical analysis revealed a significant difference in FOXA1 expression among the normal, benign, and malignant groups in the under-fifty cohort, with significantly higher expression in malignant tumors compared to benign (Figure 6d,e). Additionally, within the tumor group of all patients, a stage-specific difference in FOXA1 expression was observed (Figure 6c). However, in the overall patient group, there were no statistically significant differences in FOXA1 expression between the normal and benign groups, nor between the borderline and malignant groups. Similarly, no significant difference was detected between the benign and malignant groups. Furthermore, among tumor patients under fifty years of age, FOXA1 expression did not significantly differ among stages. In addition, tumor patients were stratified according to BRCA mutation status (negative vs. positive) and platinum sensitivity (sensitive vs. resistant) to assess FOXA1 expression levels; however, no statistically significant differences were observed in either the overall patient group or the under-fifty subgroup.

## 3. Discussion

FOXA1 has been extensively investigated across multiple cancer types. [11] In prostate cancer, FOXA1 overexpression has been shown to induce therapeutic resistance [9], whereas in osteosarcoma and colorectal cancer [15], FOXA1 knockdown reduces cell proliferation, migration, and invasion. In contrast, studies in liver cancer have demonstrated that lower FOXA1 expression is associated with increased tumor aggressiveness, and this effect appears to vary according to sex. In breast cancer, FOXA1 overexpression correlates with a better prognosis, suggesting a potential tumor-suppressive role in that context [9,10]. Collectively, these findings indicate that FOXA1 may exert distinct biological functions depending on the cancer type, sex, tissue origin, and hormonal receptor status. In addition, studies in bladder cancer and tumor-associated macrophages (TAMs) have shown that low FOXA1 expression is linked to higher infiltration of cytotoxic T lymphocytes (CTLs) and lower infiltration of M2-type TAMs [16]. These immune conditions are associated with improved chemotherapy responses. Therefore, FOXA1 expression in bladder cancer appears to influence the immune landscape of the tumor microenvironment and affect therapeutic outcomes.

In this study, we performed proliferation, migration, invasion, and wound-healing assays to determine whether FOXA1 affects the functional behavior of ovarian cancer cells. The results showed that FOXA1 knockdown significantly reduced proliferation, migration, and invasion in ovarian cancer cell lines compared with control cells. Immunohistochemical analysis of patient samples demonstrated that, in the under-fifty age group, FOXA1 expression levels differed significantly among normal, benign, and malignant tissues. Furthermore, within the malignant tumor cohort, FOXA1 expression varied significantly according to tumor stage. These findings suggest that FOXA1 promotes ovarian cancer cell proliferation, migration, and invasion—processes closely associated with metastasis—and that changes in EMT-related gene expression may contribute to this behavior. Based on these results, FOXA1 can be identified as an oncogene in ovarian cancer, highlighting its potential as a novel therapeutic target. Ovarian cancer is considered one of the representative “cold tumors,” and it is known for its limited responsiveness to immune checkpoint inhibitors [17,18]. Although the exact mechanisms underlying this poor immunogenicity remain unclear, recent studies suggest that differences in immune-cell infiltration patterns and variations in the immune microenvironment among histologic subtypes and grades contribute to the lack of response [19]. Consequently, immunotherapy has not yet achieved meaningful clinical success in ovarian cancer [20], and several phase III clinical trials involving patients with platinum-sensitive recurrent ovarian cancer (PSROC) are currently ongoing to address these limitations [21]. Within this context, our findings are noteworthy. FOXA1 inhibition decreased resistance to carboplatin and enhanced the therapeutic efficacy of combined treatment with the immune checkpoint inhibitor atezolizumab in ovarian cancer cell lines (SK-OV3 and HEYA8). However, we acknowledge that these observations were obtained using in vitro experimental systems, which do not fully recapitulate the complexity of tumor–immune interactions present in vivo. Therefore, the immunotherapeutic effects observed in this study should be interpreted with caution. This study demonstrates that FOXA1 functions as an oncogenic factor in ovarian cancer by promoting cell proliferation, migration, invasion, and EMT activation. Our data further suggest that FOXA1 may contribute to chemoresistance and influence responsiveness to immune checkpoint blockade at the cellular level. While these findings support the potential of FOXA1 as a prognostic biomarker and therapeutic target, additional in vivo and clinical studies will be required to validate its role in modulating antitumor immune responses.

Taken together, our results provide novel insight into the oncogenic role of FOXA1 in ovarian cancer and suggest that targeting FOXA1 may represent a promising strategy to overcome chemoresistance and potentially improve immunotherapeutic responsiveness. Nonetheless, future studies incorporating immune-competent in vivo models and clinical validation will be essential to fully establish the translational relevance of these findings.

## 4. Materials and Methods

### 4.1. Cell Lines and Culture

Human ovarian cancer cell line SK-OV3 (high-grade serous ovarian cancer cell line) and HEYA8 (metastatic ovarian cancer cell line) were obtained from the Korean Cell Line Bank (KCLB, Seoul, Republic of Korea). Cells were grown in RPMI 1640 (WELGENE, Gyeongsan, Republic of Korea, LM 011-01) media containing 10% FBS (Young-in Frontier, Seoul, Republic of Korea, US-FBS-500) and 1% ABS (Antibiotic-Antimycotic Solution, Corning, Corning, NY, USA, 30-004-CI) at 37 °C in a humid atmosphere containing 5% CO_2_.

### 4.2. Patients and Tumor Tissue Samples

A total of 76 case samples were used in this study (normal: 9, benign: 34, tumor: 33). Samples were collected at the time of surgery from ovarian cancer patients and hysterectomy patients, and the expression of FOXA1 was confirmed by immunohistochemical staining.

### 4.3. Real-Time Polymerase Chain Reaction

Total RNA was isolated from cell lines using the Hybrid-R™ RNA extraction kit (GeneAll, Seoul, Republic of Korea, 305-101). Reverse transcription was performed using the ReverTra Ace^®^ qPCR RT kit (Toyobo, Osaka, Japan, FSQ-201). Real-time PCR was performed using the SYBR^®^ Green Real-time PCR Master Mix kit (Toyobo, Osaka, Japan, QPK-101) and the following primer pairs: FOXA1 forward: 5-CAC TGC AAT ACT CGC CTT ACG-3 and reverse 5-TGT TTA GGA CGG GTC TGG AAT-3; and GAPDH forward: 5-TGT TCG TCA TGG GTG TGA AC-3 and reverse: 5-GCA GGG ATG ATG TTC TGG AG-3. The PCR cycle included one cycle at 95 °C for 3 min, followed by 40 cycles at 95 °C for 15 s, 60 °C for 15 s, and 72 °C for 25 s. To further investigate the expression of FOXA1 and its association with EMT (epithelial–mesenchymal transition), we performed real-time PCR using the primers listed in the table below (Table 2).

### 4.4. Small Interfering RNA (siRNA) Transfection

We seeded 2 × 10^6^ SK-OV3 and HEYA8 cells per 100 mm^2^ culture dish. Once the cells reached 50–60% confluency, the medium was changed to a serum-free RPMI 1640 medium, and the cells were cultured for 24 h. Subsequently, 20 μL of 15 μg/μL human FOXA1 small interfering RNA (siRNA, Bioneer, Daejeon, Republic of Korea) was mixed with 60 μL HiPerFect Transfection Reagent (Qiagen, Hilden, Germany, 301704) and added to the cells, and the cells were cultured for 48 h.

### 4.5. Western Blot

Cells were washed with phosphate-buffered saline (PBS) and lysed using Pro-Prep™ Protein Extraction Solution (INtRON, Seongnam, Republic of Korea, 17081). The lysates were centrifuged, and the supernatants were denatured by boiling. Protein concentrations were determined using the bicinchoninic acid (BCA) assay. Equal amounts of protein (30 µg/lane) were separated by 10% sodium dodecyl sulfate–polyacrylamide gel electrophoresis (SDS-PAGE) and transferred onto Immobilon polyvinylidene difluoride (PVDF) membranes (Millipore, Burlington, MA, USA, T831.1). The membranes were blocked with 5% skim milk for 1 h at room temperature. The membranes were incubated overnight at 4 °C with primary antibodies for FOXA1 (Santa Cruz, Dallas, TX, USA, sc-101058, 1:1000) and β-actin (Santa Cruz, Dallas, TX, USA, sc-47778, 1:1000). After washing, the membranes were incubated with a secondary antibody (Promega, Madison, WI, USA, W4021, 1:1000) for 2 h at room temperature. Protein signals were detected using enhanced chemiluminescence (ECL) Western blot detection reagents (Advansta, San Jose, CA, USA, K-12049-D50). Chemiluminescent images were captured using a chemiluminescence bioimaging system (CELLGENTEK, Daejeon, Republic of Korea, CheBi). FOXA1 protein expression levels were normalized to β-actin to ensure equal protein loading across samples.

### 4.6. Cell Proliferation Analysis

Cell proliferation was assessed using the EZ-Cytox Cell Viability Assay Kit (DoGenBio, Seoul, Republic of Korea, EZ-3000). Cells were seeded in 96-well plates in RPMI 1640 medium supplemented with 10% FBS and 1% ABS. To compare cell viability following FOXA1 knockdown and treatment with carboplatin alone or in combination with atezolizumab, the following experimental variables were applied.

Cell conditions included SK-OV3 and HEYA8 control cells and FOXA1-silenced SK-OV3 and HEYA8 cells. Drug treatment conditions included untreated controls, carboplatin monotherapy (0, 100, 120, 150, and 200 μM), and combined treatment with carboplatin (0, 100, 120, 150, and 200 μM) and atezolizumab (5 μg/mL). A total of 2 × 10^4^ cells per well were seeded and cultured for 48 h. After incubation, the culture medium was removed, and cells were treated with the indicated drugs (carboplatin and/or atezolizumab). Following 48 h of drug treatment, the medium was removed and replaced with RPMI 1640 containing 10% EZ-Cytox reagent. The plates were then incubated for 2 h at 37 °C in a humidified incubator with 5% CO_2_. Absorbance was measured at 450 nm using a microplate reader. All experiments were performed in triplicate.

### 4.7. Transwell Migration Assay

A migration assay was performed to measure the ability of cells to migrate. Using a 24-well plate containing a 6.5mm polycarbonate filter (Corning, New York, NY, USA, 3422), the transwell was added with 250 µL of RPMI 1640 and 3 × 10^5^ cells. In the bottom chamber, 750 µL of RPMI 1640 medium containing 10% FBS and 1% ABS was added. It was incubated for 48 h in a humidified incubator at 37 °C containing CO_2_. After that, the medium in the bottom chamber and the transwell chamber was removed and the chamber was washed three times using phosphate-buffered saline (PBS). To fix the cells in the membrane of the washed chamber, 4% formaldehyde was applied to the transwell and bottom chambers and reacted for 2 min. After washing 3 times with PBS, 100% methyl alcohol was applied and reacted for 10 min. Additionally, cells were stained by immersing the transwell chamber in 0.005% crystal violet solution for 2 min. Using an optical microscope, 5 sites of membrane were randomly photographed (900 × 1200 μm^2^) [22].

### 4.8. Transwell Invasion Assay

Invasion was assayed using the 24-well plate containing a 6.5 mm polycarbonate filter. The inside of the transwell was covered with serum-free RPMI 1640 medium 1:4 ratio of Matrigel dilution in RPMI 1640, and incubated for 1 h at 37 °C. 5 × 10^5^. Cells were then plated in the insert cells, and 750 µL of RPMI 1640 medium with 10% FBS and 1% ABS was added to the bottom chamber. After 72 h of incubation, cells were fixed with 4% formaldehyde and stained with 0.005% crystal violet [22].

### 4.9. Wound Healing Assay

We seeded 1 × 106 cells per well in a 12-well plate and cultured to 80% confluence. The monolayers were scratched with a 10-µL pipette tip, and the cells were washed with PBS and cultured in RPMI 1640 medium with 10% FBS and 1% ABS. The wounds were observed under a microscope and photographed at 0, 12, 24, 36, and 48 h. The percentage of wound closure fields was calculated using the Image J software (NIH, 1.52a) [22].

### 4.10. Immunohistochemical Staining for FOXA1

Paraffin-embedded patient tissue blocks sectioned at 4 µm thickness. The slides were allowed to dry for a day and were left to warm at 60 °C for an hour. For antigen retrieval, 3% H_2_O_2_ and 95 °C antigen retrieval buffer were used. For permeability, 0.2% triton solution was treated, and for blocking, 5% BSA in PBS was treated for 15 min. The 1st antibody was treated with mouse monoclonal antibody HNF-3alpha (Q-6) (Santa Cruz, Dallas, TX, USA, sc-101058, 1:100) and incubated at 4 °C overnight. Additionally, goat anti-mouse IgG (H + L), using HRP conjugate (Promega, Madison, WI, USA, W4021, 1:100), was treated as a secondary antibody for 2 h at room temperature. We stained with DAB Substrate Kit (3,3-diaminobenzidine, Vector Laboratories, Newark, CA, USA, SK-4100) and counterstained with 50% Hematoxylin for 30 s. Then, the slide was dried at 37 °C for 10 min and mounted with Eukitt^®^ Quick-hardening mounting medium (Sigma Aldrich, St. Louis, MO, USA, 03989-100).

### 4.11. Statistical Analysis

Statistical analysis was performed using SPSS 18.0 (SPSS Inc., Chicago, IL, USA). Data are expressed as mean ± SD, and statistical comparisons between two groups were conducted using Student’s *t*-test.

Cell area was quantified using ImageJ (version 1.54f; National Institutes of Health, Bethesda, MD, USA; https://imagej.net/ij/ (accessed on 7 February 2024).

## 5. Conclusions

To investigate the role of FOXA1 in ovarian cancer, we silenced FOXA1 expression using siRNA in ovarian cancer cell lines and performed functional assays, including proliferation, migration, invasion, and wound-healing analyses. FOXA1 knockdown significantly reduced these cellular functions, indicating that FOXA1 contributes to aggressive tumor-associated phenotypes. In addition, correlation analysis with EMT-related genes suggested that elevated FOXA1 expression is associated with EMT-related molecular changes linked to ovarian cancer progression.

Immunohistochemical analyses demonstrated statistically significant differences in FOXA1 expression among normal, benign, and malignant ovarian tissues, with stage-associated variation observed within the tumor group. Although these findings suggest a potential association between FOXA1 expression and disease progression, the statistical correlations observed in patient samples were limited and should be interpreted cautiously, indicating that further validation is required before FOXA1 can be established as a clinical biomarker.

Furthermore, chemoresistance assays showed that FOXA1 suppression reduced resistance to carboplatin and enhanced the anticancer effects of combined treatment with carboplatin and the immune checkpoint inhibitor atezolizumab in vitro. Given the limited efficacy of immune checkpoint inhibitors in ovarian cancer, these findings suggest that FOXA1 modulation may influence cellular sensitivity to immunotherapy. Overall, our results indicate that FOXA1 functions as an oncogenic factor in ovarian cancer and may represent a potential therapeutic target, although additional in vivo and clinical studies are needed to confirm its translational relevance.

## Figures and Tables

**Table 1:** Characteristics of the FFPE sample from patients.

	Total *n* = 76	Low *n* = 30	High *n* = 46	*p*-Value
Age, mean ± SD	53.18 ± 14.90	51.00 ± 15.19	54.61 ± 14.70	0.305
Tumor Classification, n (%)				
Normal	9 (11.8)	1 (3.3)	8 (17.4)	0.093
Benign	34 (44.7)	17 (56.7)	17 (37.0)	
Malignant tumor	ova33 (43.4)	12 (40.0)	21 (45.7)	
Stage, *n* (%)				
1	10 (30.3)	2 (16.7)	8 (38.1)	0.019
2	2 (6.1)		2 (9.5)	
3	14 (42.4)	4 (33.3)	10 (47.6)	
4	7 (21.2)	6 (50.0)	1 (4.8)	
Survival, *n* (%)				
Dead	1 (3.0)		1 (4.8)	0.320
Alive with disease	11 (33.3)	6 (50.0)	5 (23.8)	
Alive	21 (63.6)	6 (50.0)	15 (71.4)	
BRCA mutation, *n* (%)				
Negative	20 (83.3)	7 (77.8)	13 (86.7)	0.615
Positive	4 (16.7)	2 (22.2)	2 (13.3)	
Platinum, *n* (%)				
Sensitive	24 (92.3)	10 (100.0)	14 (87.5)	0.508
Resistance	2 (7.7)		2 (12.5)	

**Table 2:** Real-time PCR primer sequence.

Gene ID		Forward Primer (5′->3′)	Length	Ensembl Genome
E-cadeherin	Forward	AACTCCAGGCTAGAGGGTCA	21	ENSG00000039068
	Reverse	TCACAGGTGCTTTGCAGTTC	21	
N-cadeherin	Forward	GAGACTTGCGAAACTCCAGAC	21	ENSG00000170558
	Reverse	CATTAAGCCGAGTGATGGTCC	21	
SNAIL1	Forward	CCCCAATCGGAAGCCTAACTA	21	ENSG00000124216
	Reverse	ACAGAGTCCCAGATGAGCATT	21	
VIM	Forward	ACTTTTCCTCCCTGAACCTGA	21	ENSG00000026025
	Reverse	TTCAAGGTCATCGTGATGCTG	21	
ZEB1	Forward	TCCAGGAAGAACCCTTGAACT	21	ENSG00000148516
	Reverse	CTGGGCAGTGACTGTAGGTAT	21	
COLA1	Forward	GAATGGAGATGATGGGGAAGC	21	ENSG00000108821
	Reverse	ACCATCCAAACCACTGAAACC	21	

## Data Availability

The data presented in this study are available upon reasonable request from the corresponding author. The data are securely stored at the Future Innovative Medical Research Center, Soonchunhyang University Cheonan Hospital. Due to ethical and privacy restrictions related to patient information, the data are not publicly available.
